# West Nile Virus Infection in Belgian Traveler Returning from Greece

**DOI:** 10.3201/eid1904.121594

**Published:** 2013-04

**Authors:** Lieselotte Cnops, Anna Papa, Frideriki Lagra, Philippe Weyers, Kathleen Meersman, Nicolas Patsouros, Marjan Van Esbroeck

**Affiliations:** National Reference Center for WNV and Arboviruses–Institute of Tropical Medicine, Antwerp, Belgium (L. Cnops, K. Meersman, M. Van Esbroeck);; National Reference Laboratory for Arboviruses–Aristotle University Medical School, Thessaloniki, Greece (A. Papa;; Kavala General Hospital, Kavala, Greece (F. Lagra);; Saint-Jean Hospital, Brussels, Belgium (P. Weyers, N. Patsouros)

**Keywords:** West Nile virus, encephalitis, traveler, Greece, viruses, vector-borne infections

**To the Editor:** West Nile virus (WNV) is an arthropod-borne virus that is transmitted to humans by mosquitos, primarily of the genus *Culex.* Most human infections are asymptomatic. Clinical symptoms occur in ≈20% of case-patients and include fever, headache, and myalgia; <1% of WNV infections develop into severe neuroinvasive disease (*1*).

The virus was discovered in 1937 in the West Nile district of Uganda. WNV is endemic to parts of Africa, Europe, Asia, and the Middle East, and since its introduction in New York in 1999, in North America. In Eurasia, human WNV infections were first reported in Israel and France during the 1950s–1960s, and the first major outbreak in Romania occurred in 1996 (*1*). The disease emerged recently in Greece; a large outbreak in 2010 caused neuroinvasive disease in 197 patients, of whom 33 died (*2*). Since 2010, occasional and local epidemics have been ongoing in Greece, Italy, Romania, Hungary, Spain, and the Balkans (*3*,*4*).

Clinical diagnosis may be difficult because WNV infections resemble other (arbo)viral diseases. Laboratory diagnosis relies primarily on serologic testing. Reverse transcription PCR (RT-PCR) can be used to detect viral RNA during the acute phase of the disease, but its use is hampered by the patient’s low-level and transient viremia (*1*).

We here describe a confirmed case of WNV encephalitis imported by a traveler returning from Greece. A 73-year-old Belgian woman, who had a medical history of lymphoma, traveled to Kavala city (Macedonia, Greece). On August 14, 2012, she sought treatment at the Kavala General Hospital with a 6-day history of fever, headache, malaise, nausea, confusion, decline of consciousness, and neck stiffness. Results of laboratory testing on admission demonstrated an increased leukocyte count (9,670/µL; 80% neutrophils) and lactate dehydrogenase level (522 IU/L), a low C-reactive protein level (0.7 mg/dL), and hyponatremia (131 mEq/L). Cerebrospinal fluid (CSF) testing showed 90 cells/µL (79% lymphocytes) and glucose and protein levels of 72 and 100.9 mg/dL, respectively. Serum obtained on August 15 was sent to the national reference laboratory at Aristotle University (Thessaloniki, Greece), and IgM against WNV was detected by ELISA (WNV IgM Capture DxSelect and IgG DxSelect; Focus Diagnostics, Cypress, CA, USA). IgG was absent. On the second day of hospitalization, the patient exhibited seizures (speech arrest); she was given phenytoin (1/2 amp 3×/day intravenously). On August 18, the patient was transferred to a private hospital. Further treatment included intravenous fluid, antipyretics, antimicrobial drugs, mannitol, and oxygen. On August 30, she was returned by plane to Belgium.

CSF obtained 26 days after symptom onset and serum obtained 29 days after symptom onset were sent to the Institute of Tropical Medicine (Antwerp, Belgium) because of its function as a national reference center for Belgium. IgM and IgG against WNV were detected in both samples by ELISA (Focus Diagnostics) (Table). Immunofluorescence assays on serum revealed IgM against WNV only and IgG against West Nile, dengue, yellow fever, and Japanese encephalitis viruses, with the strongest reaction against WNV (Flavivirus Mosaic 1; Euroimmun, Lübeck, Germany). Real-time RT-PCR (adapted from [5]) on the serum demonstrated a weak positive signal. Repeated RNA extraction and RT-PCR were confirmative (Table). Sequencing of the RT-PCR product confirmed the detection of WNV. Although the product was short (116 bp), it was highly suggestive of WNV, lineage 2. Flemish regional authority in Belgium, national authorities (both in Belgium and Greece), and European health authorities were notified of the imported case of WNV encephalitis. According to the case definition of the European Center for Disease Prevention and Control, Stockholm, Sweden, the patient met the laboratory criteria of having a confirmed case.

**Table:** Laboratory results confirming WNV infection of 73-year-old woman, Greece, 2012*†

Sample	Date	RT-PCR (C_t_ value)	WNV ELISA IgM (ratio)	WNV ELISA IgG (ratio)	Flavi IFAT IgM	Flavi IFAT IgG
Serum	Aug 15	Positive (45.47)	Positive (25)	Negative	ND	ND
CSF	Sep 3	ND	Positive (5.16)	Positive (2.21)	ND	ND
Serum	Sep 6	Positive (42.87)‡	Positive (4.76)	Positive (2.63)	WNV positive	WNV positive§

*WNV, West Nile virus; RT-PCR, reverse transcription PCR; Ct, cycle threshold; Flavi, flavivirus; IFAT, indirect fluorescent antibody technique; ND, not done; CSF, cerebrospinal fluid. †The ELISA is positive if ratio >1.1 for IgM and >1.5 for IgG. The cutoff value for IFAT is 1/10 for both IgG and IgM.  ‡Sequencing revealed a 116-bp sequence perfectly matched to the WNV amplicon and is highly suggestive for WNV lineage 2 on the basis of the presence of 2 specific nucleotides.  §Strongest signal for WNV, weak signal for other flaviviruses (Japanese encephalitis virus, dengue viruses 1–4, yellow fever virus).

To date, autochthonous WNV infections have not been reported in Belgium, although the presence of the mosquito vector provides a potential risk for transmission (*6*). This WNV infection was acquired in Greece (a leading travel destination for tourists from Belgium), specifically in the Kavala region, which was highly affected by WNV in 2012. The lineage responsible for the WNV encephalitis was identified as lineage 2, the currently circulating strain in Greece (*7*). Our report highlights the need for physicians and laboratory staff to be aware of imported WNV infections originating from southeastern Europe, especially Greece and its neighboring countries, where recent and recurrent outbreaks have occurred (*3*,*4*).

Special attention should be given to immunosuppressed and elderly patients who are at higher risk of acquiring neuroinvasive disease. The 73-year-old patient described here was unconscious when she arrived in Belgium. After a short period of relative improvement (more reactive and cooperative), her condition deteriorated, and she died on November 23, 2012. The detection of viral RNA 29 days after symptom onset was surprising but might be explained by the immunocompromised status of the patient. Several studies have reported persistent WNV RNA for 30 days, 77 days, and even years after the symptom onset in serum, CSF, and urine, respectively (*8*–*10*), and a prolonged period of viremia in immunocompromised patients (*9*).
